# Efficacy and Safety of Xuebijing Injection Combined With Ulinastatin as Adjunctive Therapy on Sepsis: A Systematic Review and Meta-Analysis

**DOI:** 10.3389/fphar.2018.00743

**Published:** 2018-07-24

**Authors:** Guochao Chen, Yanyan Gao, Yue Jiang, Fei Yang, Shuangshuang Li, Di Tan, Qun Ma

**Affiliations:** Department of Pharmacy of Chinese Materia Medica, School of Chinese Materia Medica, Beijing University of Chinese Medicine, Beijing, China

**Keywords:** xuebijing injection, ulinastatin, sepsis, systematic review, meta-analysis, efficacy, safety

## Abstract

**Background:** Xuebijing injection (XBJ), transforming from Xuefuzhuyu decoction, is the only Chinese medicine injection approved for sepsis. XBJ and ulinastatin (UTI) combination therapy is supposed to be beneficial for sepsis patients. To fill the gap between the lack of evidence for the efficacy of combination therapy and its increasing application among patients, an extensive meta-analysis was performed.

**Methods:** Eight databases were searched to identify randomized controlled trials (RCTs) comparing XBJ plus UTI with UTI alone in treating sepsis from inception to February 5, 2018. Data extraction and methodological quality assessment of the included RCTs were implemented by two investigators independently. All data were synthesized and analyzed utilizing Review Manager 5.3.

**Results:** Seventeen RCTs with a total of 1,247 participants corresponded with the inclusion criteria of our study. The findings reflected that in comparison to single UTI, XBJ and UTI combination therapy could significantly lower 28-day mortality (RR = 0.54, 95% CI [0.39, 0.73], *P* < 0.0001), shorten duration of mechanical ventilation (SMD = −1.13, 95% CI [−1.30, −0.95], *P* < 0.00001), reduce length of ICU stay (SMD = −0.84, 95% CI [−1.00, −0.67], *P* < 0.00001), and decrease APACHE II score (SMD = −1.09, 95% CI [−1.49, −0.69], *P* < 0.00001). Additionally, XBJ plus UTI had superiority over single UTI in lowering PCT levels (SMD = −1.61, 95% CI [−2.23, −0.98], *P* < 0.00001), and improving inflammatory cytokines—IL-6 and TNF-α levels (SMD = −1.45, 95% CI [−1.71, −1.19], *P* < 0.00001; SMD = −1.11, 95% CI [−1.42, −0.80], *P* < 0.00001). Moreover, CRP, hs-CRP, and LPS levels were remarkably reduced by XBJ plus UTI compared with UTI alone (SMD = −1.50, 95% CI [−2.00, −1.00], *P* < 0.00001; SMD = −1.31, 95% CI [−1.70, −0.93], *P* < 0.00001; SMD = −1.17, 95% CI [−1.42, −0.92], *P* < 0.00001). Three studies involving 14 patients reported the occurrences of adverse events.

**Conclusions:** Comparing with UTI alone, XBJ and UTI combination therapy appeared to be more effective for sepsis. However, owing to the limitations of this meta-analysis, additional RCTs with higher-quality and more rigorous design are needed to confirm our findings.

## Introduction

Sepsis is a complicated clinical syndrome characterized by excessive and uncontrolled host's systemic inflammatory response to infection (Liu et al., 2017). The mechanism of sepsis is not completely known, although we know systemic inflammatory reaction contributes to its morbidity in complex and multiple pathways (Remick, 2007). Severe sepsis, with an extremely high incidence and mortality in critically ill and elderly patients, is frequently accompanied by multiple organ dysfunction syndyome (MODS) and even death (Bone et al., 1992; Liu et al., 2017). In the United States, more than 1.5 million cases of sepsis occur annually and was estimated to cost $24.3 billion in 2007, which places a substantial burden on the healthcare system (Lagu et al., 2012; Seymour et al., 2017). Despite rapid progresses achieved in clinical treatment over the past decades, sepsis still has a high intensive care units (ICU) admission rate and is a leading cause of death in many ICU (Angus et al., 2001; Backer and Dorman, 2017). Up until now, precautionary measures, specific medications, and management strategies for the control of sepsis are quite limited.

Ulinastatin (UTI), a broad-spectrum protease inhibitor separated and purified from human urine, was originally applied to acute pancreatitis patients (Tsujino et al., 2005). With the deepening of research, clinical researchers discovered it has an inhibitory effect not only on various protease activities, such as trypsin, kallikrein, plasmin, thrombin and so forth, but also on the release of inflammatory cytokines induced by adverse immunostimulation (Linder and Russell, 2014). On the basis of aforementioned properties, UTI has been widely applied for the treatment of sepsis in Asia (Zhang et al., 2014).

Xuebijing injection (XBJ), a Chinese medicine preparation, is transformed from a classical formula—Xuefuzhuyu decoction—under the guidance of traditional Chinese medicine therapeutic principle “bacteria and bacterial toxin treated simultaneously” (Zuo et al., 2017). It is made from five Chinese herb extracts including Radix Angelicae Sinensis, Rhizoma Chuanxiong, Radix Paeoniae Rubra, Radix Salviae Miltiorrhizae, and Flos Carthami, which work together to implement the effects of activating blood circulation to dissipate blood stasis and cooling blood to remove toxic substances. XBJ was approved by Food and Drug Administration (FDA) of China in 2004, aiming directly at the treatment of sepsis and MODS (He et al., 2013; Yin and Li, 2014). Pharmacological studies manifested XBJ blocks the progression of sepsis through anti-bacteria, anti-inflammation and anti-endotoxin, which is an effective agent for improving survival rate (Ma et al., 2009).

Recently, an increasing number of clinical trials suggested that XBJ and UTI combination therapy had beneficial implications on sepsis patients' conditions and prognoses (Liao et al., 2014; Wang et al., 2014; Jiang et al., 2015), however, no definite conclusion was drawn on this. To provide believable and solid evidence whether XBJ combined with UTI can improve the efficacy of UTI for sepsis, this meta-analysis was performed by systematically evaluating the efficacy and safety of XBJ plus UTI compared with UTI alone.

## Methods

### Data sources and filtration strategy

All randomized controlled trials (RCTs) comparing XBJ plus UTI with UTI alone in treating sepsis were retrieved. Eight databases, including Cochrane Library, PubMed, Embase, Web of Science, China Science and Technology Journal Database (VIP), China National Knowledge Infrastructure (CNKI), Chinese Biomedical Database (CBM), and WanFang Database, were searched to identify all relevant publications from inception to February 5, 2018. Search terms included “Xuebijing injection”, “ulinastatin”, and “sepsis”. The following search strategy was utilized and modified into various forms to suit all databases: “Xuebijing injection” [Title/Abstract] AND “ulinastatin” [Title/Abstract] AND “sepsis” [Title/Abstract]. References of retrieved literatures and reviews were checked to collect potentially relevant studies.

### Inclusion criteria

Studies conformed to the following items could be involved in this meta-analysis: (1) Study type: RCTs published in English or Chinese comparing XBJ plus UTI with UTI alone for the treatment of sepsis. (2) Participants: All patients were diagnosed as sepsis in accordance with internationally recognized diagnostic criteria proposed by the American College of Chest Physicians/Society of Critical Care Medicine (ACCP/SCCM) Consensus Conference in 1991 or International Sepsis Definitions Conference in 2001 (Bone et al., 1992; Levy et al., 2003). No restrictions were set on age, race, gender, or disease severity. (3) Interventions: Both the experimental and control groups received conventional therapies, on the basis of this, the experimental group was administered XBJ combined with UTI, while the control group was administered UTI alone. Conventional therapies were implemented according to international guidelines for management of sepsis, which include controlling the source of infection, empiric antimicrobial therapy, hemodynamic support, mechanical ventilation, nutritional support, and so on (Rhodes et al., 2017). No limitations were set on dosages and courses of the treatment. (4) Outcomes: One or more outcome indicators of the following must be involved: 28-day mortality, duration of mechanical ventilation, length of ICU stay, acute physiology and chronic health evaluation (APACHE) II score, serum levels of inflammatory cytokines, procalcitonin (PCT), C-reactive protein (CRP)/high-sensitivity C-reactive protein (hs-CRP), and lipopolysaccharide (LPS).

### Exclusion criteria

The criteria for exclusion were as follows: (1) duplicate literatures, reviews, commentaries, meta-analyses, animal and cell experiments. (2) Full texts of the studies could not be obtained. (3) Data of the articles was statistically flawed. (4) Any other specific medicines or interventions were involved in the experimental group or control group. (5) Patients with complications, such as serious heart, liver, lung, kidney, coagulation and other organ or system diseases, HIV infection, malignant tumors, connective tissue diseases; discharged or died within 72 h of treatment. (6) As for duplicate publications with the similar authors and results, the one that had larger sample size and more complete data was included.

### Study selection

Two researchers (GC, YG) independently browsed the literature titles and abstracts to rule out studies that did not meet the criteria established above. Full texts of the remaining potential studies were obtained to validate their inclusion or not. When discrepancies occurred, they were resolved by discussing to reach an consensus or consultation with a third party (QM).

### Data extraction and quality assessment

Two researchers (GC, YG) independently performed data extraction of each identified study for the following information with a pre-specified electronic table: first author, publication year, age, sample sizes of the experimental and control groups, intervention measures, dosages, courses of treatment, outcome measures, and the number of adverse events (AEs).

The Cochrane Risk of Bias Assessment Tool was utilized to assess methodological quality of the identified studies, which contains 7 aspects: (1) random sequence generation, (2) allocation concealment, (3) blinding of participants and personnel, (4) blinding of outcome assessment, (5) incomplete outcome data, (6) selective reporting, and (7) other bias (Higgins et al., 2011). The quality of each item was assessed as high risk, uncertain risk, or low risk. When researchers had uncertainty about the information of treatment and methodology, the original authors were contacted via telephone or e-mail to acquire additional information. If there were no responses, the trials were ruled out.

### Statistical analysis

This meta-analysis was conducted utilizing Review Manager 5.3 (Cochrane Collaboration, Oxford, UK). For dichotomous variables, outcomes were expressed as risk ratio (RR) along with 95% confidence intervals (CI), while for continuous variables, mean difference (MD) or standard mean difference (SMD) together with 95% CI were calculated. Chi-square test and *I*^2^ test were applied to reflect statistical heterogeneity among pooled RCTs (Higgins et al., 2003). *P* ≥ 0.1 and *I*^2^ ≤ 50% was deemed as acceptable homogeneous data and a fixed-effects model was carried out, otherwise a random-effects model was performed due to data with significant heterogeneity (*P* < 0.1 and *I*^2^ > 50%). Where possible, a funnel plot would be utilized to assess publication bias. Besides, to test the robustness of the outcome, a sensitivity analysis of 28-day mortality was performed using STATA 12.0 (Stata Corp, College Station, TX).

## Results

### Search results

**Figure 1:**
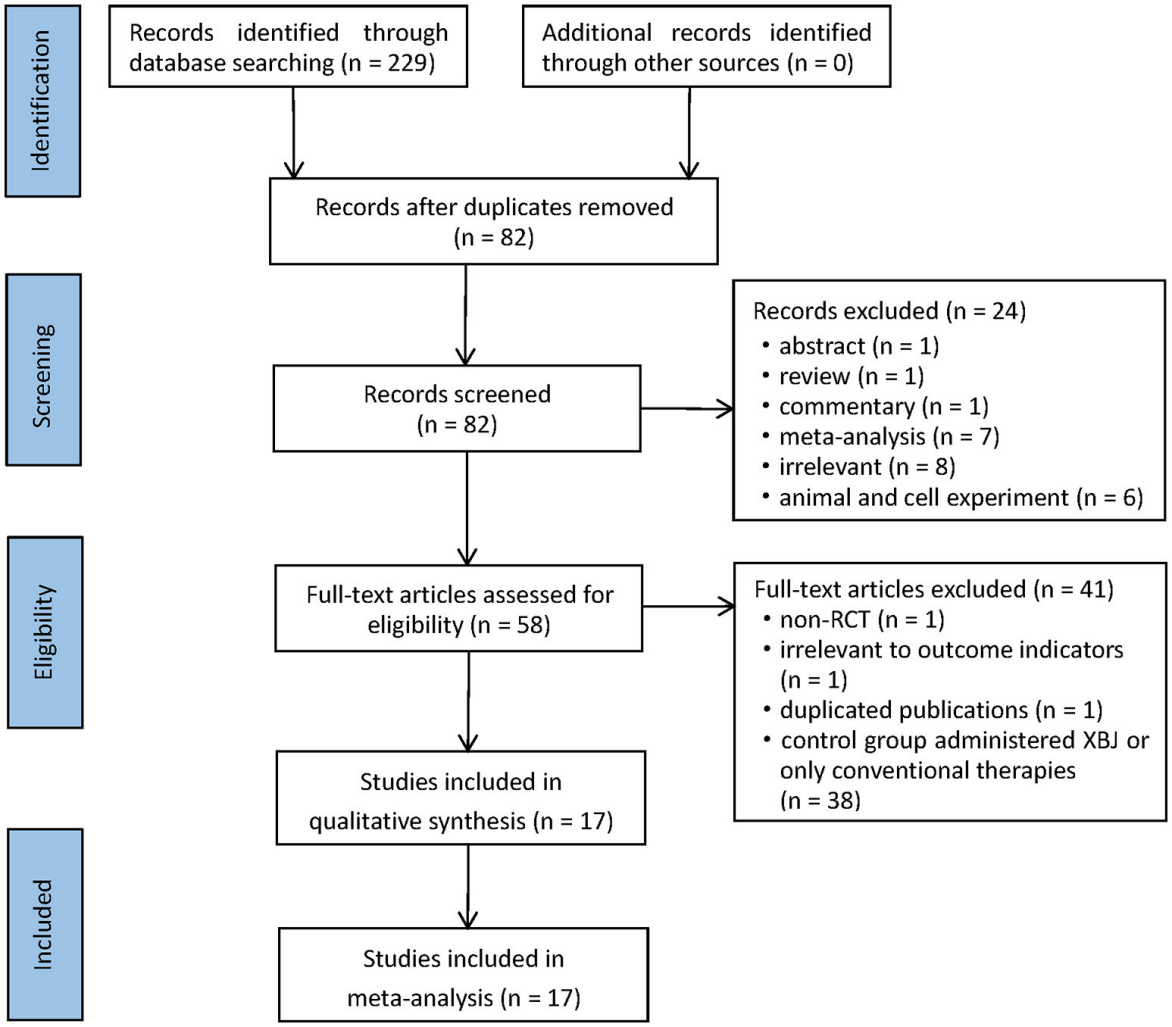
Flow chart of literature search.

In accordance with the search strategy, 229 potentially relevant records were retrieved in the initial search. After browsing titles and abstracts, 147 articles were removed due to duplicates, and 58 literatures were selected for full-text reading. Ultimately, 17 RCTs corresponding with the inclusion criteria were included in this meta-analysis (Mao et al., 2008; Sun et al., 2010; Ye and Wu, 2010; Abuli et al., 2013; Jiang and Mao, 2013; Wang, 2013; Zhao and Liu, 2013; Zhou and Fang, 2013; Li, 2015, 2016; Ji et al., 2016; Shan, 2016; Bian et al., 2017; Chen, 2017; Chen et al., 2017; Li and Jia, 2017; Lu et al., 2017). A detailed flowchart that presented the process of selection was shown in Figure 1.

### Characteristics of included studies

The 17 included studies, consisting of a total of 1,247 participants, were published in Chinese academic journals between 2008 and 2017. On the basis of conventional therapies, 621 participants in the control group and 626 participants in the experimental group were administered single UTI and XBJ combined with UTI respectively. The doses of UTI ranged from 100 to 900 KU, with the frequency ranging from once a day to thrice a day. When it came to XBJ, the doses administered were 40, 50, or 100 ml, with the frequency ranging from once a day to thrice a day. Courses of treatment varied from 7 to 14 days, the vast majority of which were 7 days. The identified RCTs and their primary information are listed in Table 1.

**Table 1:** Characteristics of the included studies.

**Study ID**	**Age**	**Sample size EG/CG**	**Interventions**		**Course of treatment (days)**	**Outcome measures**	**AEs**
			**EG**	**CG**			
Mao et al., 2008	EG:50.1 ± 9.6 CG:51.5 ± 11.5	57/57	XBJ 100ml Q12h + UTI 200KU Q12h + CT	UTI 200KU Q12h + CT	7	(1),(2),(3),(5),(6),(7),(10)	None
Sun et al., 2010	–	20/20	XBJ 100ml Q12h + UTI 200KU Q12h + CT	UTI 300KU Q12h + CT	10	(2),(3)	None
Ye and Wu, 2010	20–58	27/23	XBJ 100ml Q12h + UTI 200KU Bid + CT	UTI 200KU Bid + CT	7	(4),(5)	Unclear
Zhou and Fang, 2013	43.47 ± 1.38	61/61	XBJ 100ml/d + UTI 900KU/d + CT	UTI 900KU/d + CT	14	(5),(6),(7)	Unclear
Zhao and Liu, 2013	27–83	44/44	XBJ 100ml Bid + UTI 200KU Bid + CT	UTI 200KU Bid + CT	7	(1),(2),(3),(4),(5),(6),(7),(10)	CG 2
Abuli et al., 2013	20–60	15/15	XBJ 50ml Q12h + UTI 300KU Q12h + CT	UTI 300KU Q12h + CT	7	(2),(3)	None
Jiang and Mao, 2013	EG:49.5 ± 11.2 CG:49.3 ± 11.5	43/43	XBJ 50ml Bid + UTI 200KU Tid + CT	UTI 200KU Tid + CT	7	(1),(2),(3),(4),(5),(6),(7),(10)	EG 3, CG 2
Wang, 2013	EG:45.4 ± 8.5 CG:45.9 ± 7.6	20/20	XBJ 40ml Bid + UTI 200KU Q8h + CT	UTI 200KU Q8h + CT	7	(3),(4),(5),(6),(8)	None
Li, 2015	23–62	40/40	XBJ 50ml Bid + UTI 300KU Bid + CT	UTI 300KU Bid + CT	7	(2),(3)	None
Ji et al., 2016	EG:55.9 ± 8.3 CG:56.4 ± 8.8	30/30	XBJ 50ml Bid + UTI 200KU Bid + CT	UTI 200KU Bid + CT	7	(1),(2),(3),(4),(6),(7),(8)	Unclear
Shan, 2016	EG:43.1 ± 9.6 CG:41.8 ± 8.9	35/35	XBJ 50ml Bid + UTI 200KU Bid + CT	UTI 200KU Bid + CT	7	(6),(7),(9)	None
Li, 2016	EG:37.0 ± 10.8 CG:36.7 ± 10.9	40/40	XBJ 100ml Bid + UTI 100KU Bid + CT	UTI 100KU Bid + CT	7	(1),(2),(3),(4),(6),(7),(8)	Unclear
Lu et al., 2017	EG:42.3 ± 5.3 CG:42.5 ± 5.5	49/49	XBJ 50ml Bid + UTI 200KU Bid + CT	UTI 200KU Bid + CT	7	(4)	None
Li and Jia, 2017	EG:46.2 ± 10.3 CG:47.4 ± 10.8	54/54	XBJ 50ml Bid + UTI 200KU Bid + CT	UTI 200KU Bid + CT	7	(5)	Unclear
Chen et al., 2017	EG:68.7 ± 4.4 CG:67.6 ± 5.4	30/30	XBJ 50ml Tid + UTI 200KU Bid + CT	UTI 200KU Bid + CT	14	(5),(7),(9)	EG 4, CG 3
Chen, 2017	EG:33.4 ± 5.7 CG:33.0 ± 5.5	35/34	XBJ 100ml Bid + UTI 200KU Bid + CT	UTI 200KU Bid + CT	10	(1),(7)	Unclear
Bian et al., 2017	EG:39.2 ± 2.4 CG:38.7 ± 2.1	26/26	XBJ 50ml Tid + UTI 100KU Tid + CT	UTI 100KU Tid + CT	10	(6),(8)	Unclear

*EG, Experimental group; CG, Control group; XBJ, Xuebijing injection; UTI, Ulinastatin; CT, Conventional therapies; Outcome measures: (1), 28-Day mortality; (2), Duration of mechanical ventilation; (3), Length of ICU stay; (4), APACHE II score; (5), Procalcitonin, (6), Interleukin-6; (7), Tumor necrosis factor-α; (8), C-reactive protein; (9), High-sensitivity C-reactive protein; (10), Lipopolysaccharide; AEs, Adverse events*.

### Risk of bias in included studies

**Figure 2:**
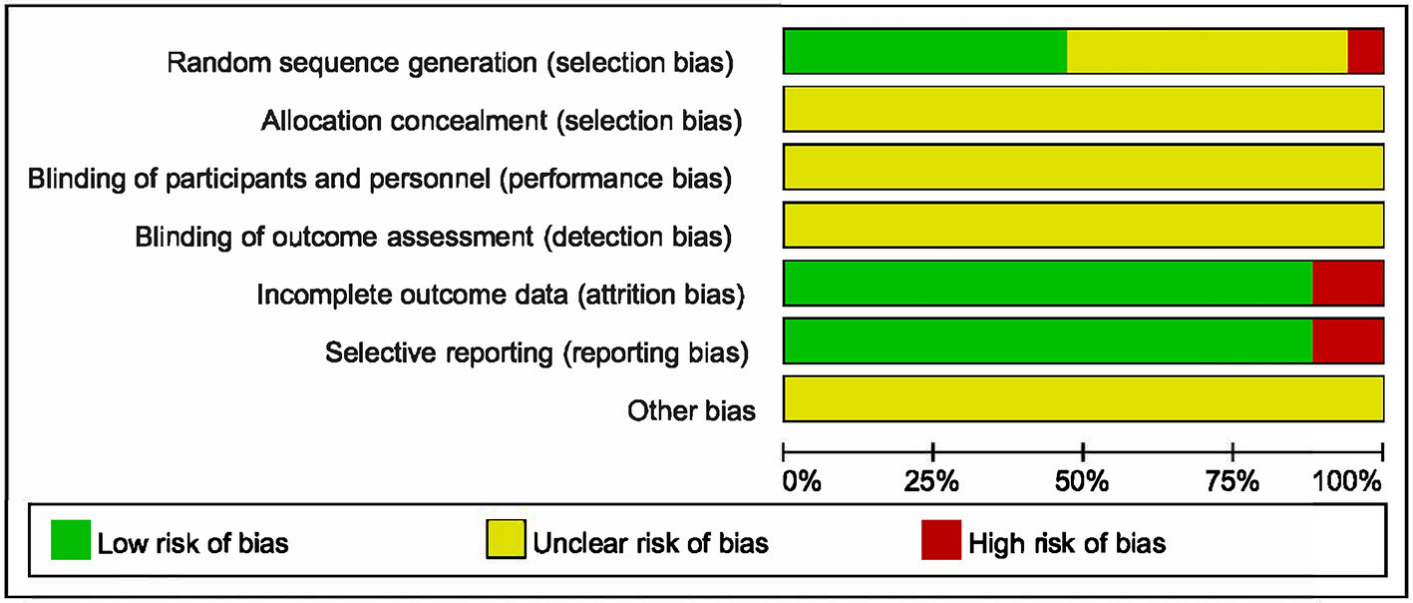
Risk of bias graph.

Methodological quality of the 17 identified RCTs was assessed in accordance with the Cochrane Risk of Bias Assessment Tool. All included RCTs claimed to have adopted random allocation, while only six of them utilized random digital table (Wang, 2013; Shan, 2016; Bian et al., 2017; Chen et al., 2017; Li and Jia, 2017; Lu et al., 2017), and two utilized softwares to generate random numbers (Mao et al., 2008; Jiang and Mao, 2013). Allocation concealment, blinding of participants and personnel along with blinding of outcome assessment were assessed as unclear risk, because none of the 17 RCTs made descriptions of them. Two of the 17 trials reflected high risk of incomplete outcome and selective reporting (Sun et al., 2010; Abuli et al., 2013). Furthermore, the risk of other bias was assessed as unclear (Figure 2).

### Meta-analyses results

#### 28-day mortality

**Figure 3:**
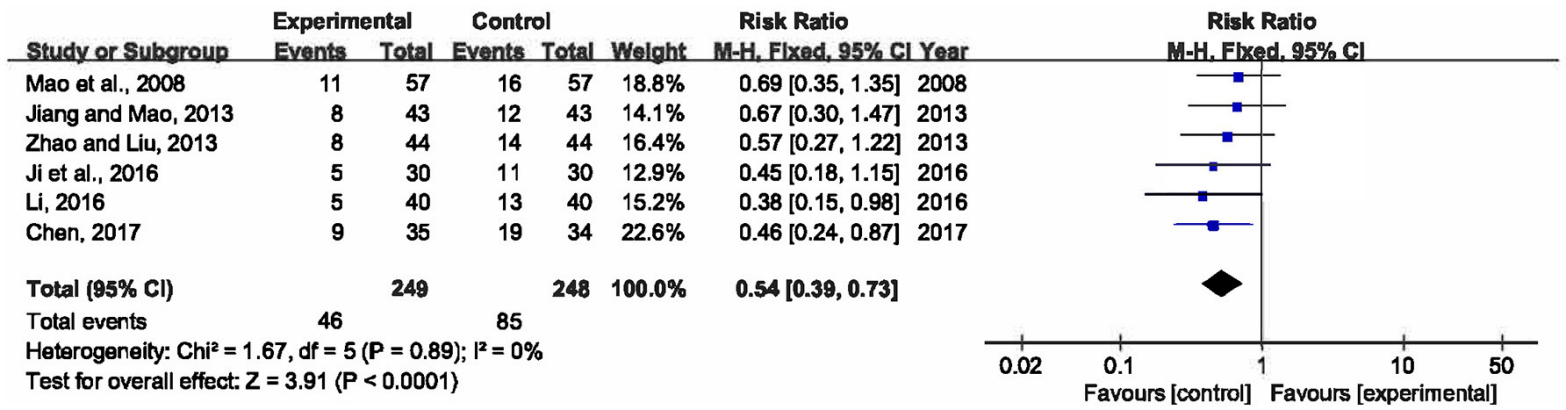
Forest plot of 28-day mortality in sepsis patients treated with XBJ+UTI therapy and UTI alone.

Six researches involving 497 participants compared mortality within 28 days between XBJ plus UTI group and single UTI group (Mao et al., 2008; Jiang and Mao, 2013; Zhao and Liu, 2013; Ji et al., 2016; Li, 2016; Chen, 2017). No significant heterogeneity was detected across the six included studies (*P* = 0.89, *I*^2^ = 0%), thus a fixed-effects model was adopted. Analysis of the pooled data manifested that XBJ plus UTI was associated with a significant lower 28-day mortality than single UTI (RR = 0.54, 95% CI [0.39, 0.73], *P* < 0.0001) (Figure 3).

### Duration of mechanical ventilation

**Figure 4:**
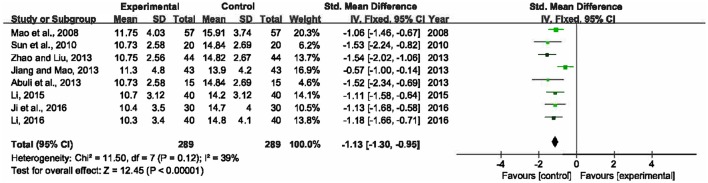
Forest plot of duration of mechanical ventilation in sepsis patients treated with XBJ+UTI therapy and UTI alone.

There were eight trials measured duration of mechanical ventilation after the treatment (Mao et al., 2008; Sun et al., 2010; Abuli et al., 2013; Jiang and Mao, 2013; Zhao and Liu, 2013; Li, 2015, 2016; Ji et al., 2016). Heterogeneity among the studies was moderate (*P* = 0.12, *I*^2^ = 39%). This meta-analysis, applying a fixed-effects model, showed that compared to single UTI, XBJ combined with UTI could significantly shorten the duration that patients had to be on a mechanical ventilator by 1.13 days (SMD = −1.13, 95% CI [−1.30, −0.95], *P* < 0.00001) (Figure 4).

### Length of ICU stay

**Figure 5:**
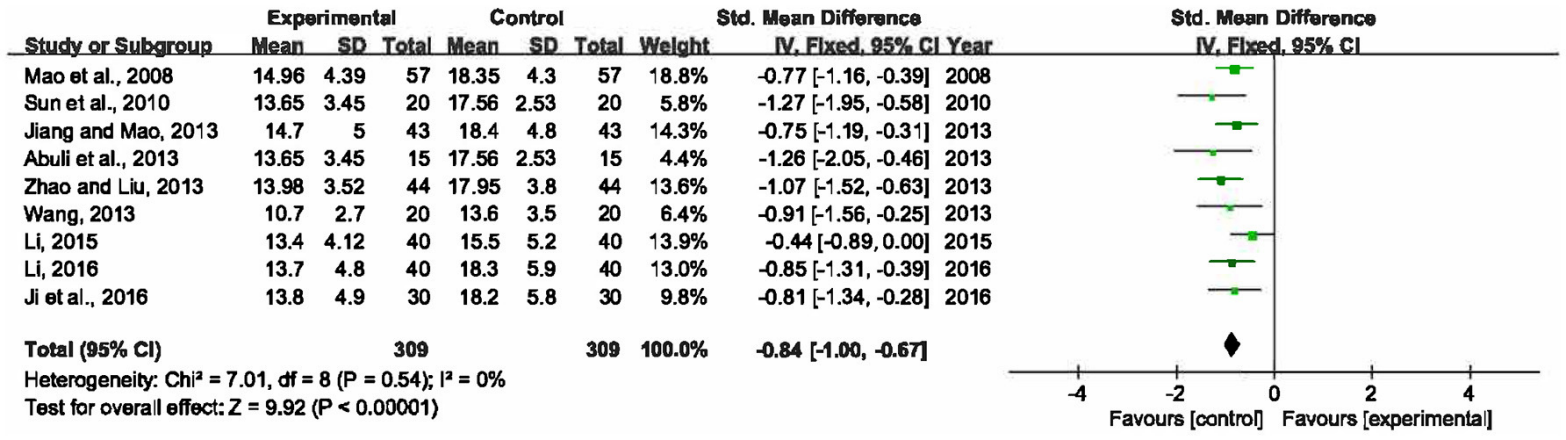
Forest plot of length of ICU stay in sepsis patients treated with XBJ+UTI therapy and UTI alone.

The length of ICU stay was reported in 9 RCTs with 618 participants enrolled (Mao et al., 2008; Sun et al., 2010; Abuli et al., 2013; Jiang and Mao, 2013; Wang, 2013; Zhao and Liu, 2013; Li, 2015, 2016; Ji et al., 2016). A fixed-effects model was selected for this meta-analysis due to no heterogeneity among the trials (*P* = 0.54, *I*^2^ = 0%). The result revealed that XBJ plus UTI group had an advantage over single UTI group in reducing the length of ICU stay by 0.84 day (SMD = −0.84, 95% CI [−1.00, −0.67], *P* < 0.00001) (Figure 5).

### APACHE II score

**Figure 6:**
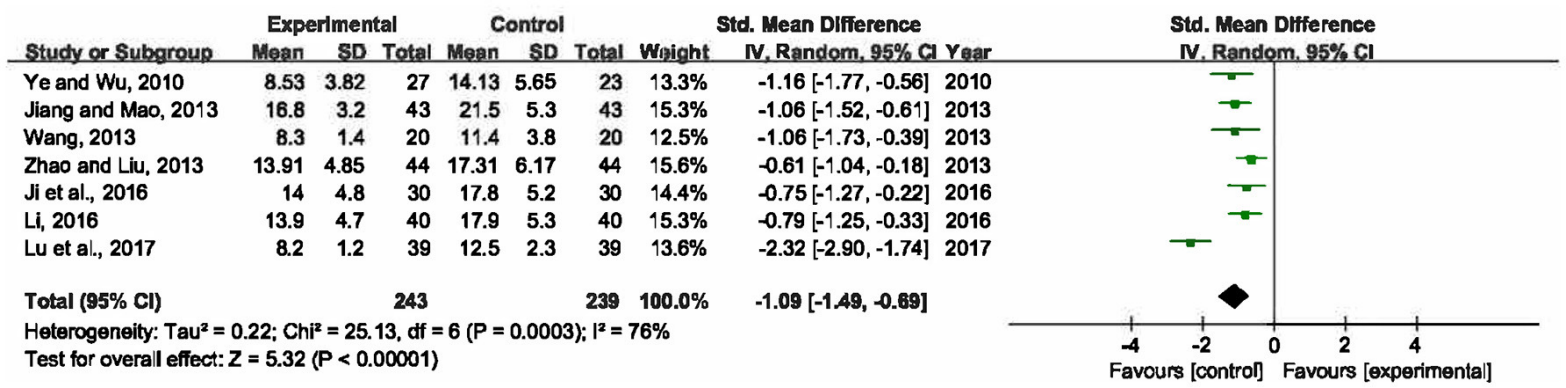
Forest plot of APACHE II score in sepsis patients treated with XBJ+UTI therapy and UTI alone.

Seven trials involving 482 participants calculated APACHE II score (Ye and Wu, 2010; Jiang and Mao, 2013; Wang, 2013; Zhao and Liu, 2013; Ji et al., 2016; Li, 2016; Lu et al., 2017). Heterogeneity of these trials was substantial (*P* = 0.0003, *I*^2^ = 76%), therefore, a random-effects model was utilized. The pooled analysis demonstrated that statistically significant difference was presented between XBJ plus UTI group and single UTI group, which meant XBJ combined with UTI was superior to single UTI in terms of ameliorating APACHE II score (SMD = −1.09, 95% CI [−1.49, −0.69], *P* < 0.00001) (Figure 6).

### Serum levels of PCT

**Figure 7:**
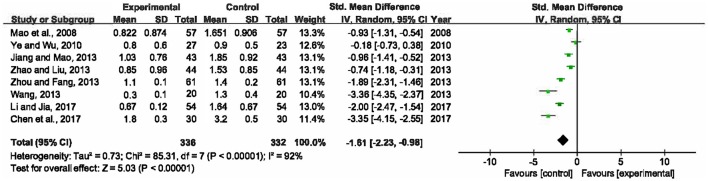
Forest plot of serum PCT levels in sepsis patients treated with XBJ+UTI therapy and UTI alone.

Eight included RCTs (Mao et al., 2008; Ye and Wu, 2010; Jiang and Mao, 2013; Wang, 2013; Zhao and Liu, 2013; Zhou and Fang, 2013; Chen et al., 2017; Li and Jia, 2017), involving 668 participants, measured levels of PCT in serum. Since significant heterogeneity was detected among the trials (*P* < 0.00001, *I*^2^ = 92%), a random-effects model was adopted. The result signified that there was statistically significant difference between XBJ plus UTI group and single UTI group (SMD = −1.61, 95% CI [−2.23, −0.98], *P* < 0.00001), so a combination of XBJ and UTI could significantly lower PCT levels than single UTI (Figure 7).

### Serum levels of inflammatory cytokines

Inflammatory cytokines, including interleukin-6 (IL-6) and tumor necrosis factor-α (TNF-α), were measured in 11 RCTs (Mao et al., 2008; Jiang and Mao, 2013; Wang, 2013; Zhao and Liu, 2013; Zhou and Fang, 2013; Ji et al., 2016; Li, 2016; Shan, 2016; Bian et al., 2017; Chen, 2017; Chen et al., 2017). The random-effects model was utilized for substantial heterogeneity among the studies (*P* = 0.02, *I*^2^ = 57%; *P* = 0.0002, *I*^2^ = 74%). As shown in Table 2, co-administration of XBJ with UTI was superior to single UTI in reducing serum levels of inflammatory cytokines—IL-6 and TNF-α (SMD = −1.45, 95% CI [−1.71, −1.19], *P* < 0.00001; SMD = −1.11, 95% CI [−1.42, −0.80], *P* < 0.00001).

**Table 2:** Meta-analysis of inflammation indexes.

**Outcomes**	**RCTs**	**Heterogeneity**	**Model**	**SMD [95% CI]**	***Z***	***P***
IL-6	9	*P* = 0.02, *I*^2^ = 57%	Random-effects model	−1.45 [−1.71, −1.19]	10.98	0.00001
TNF-α	9	*P* = 0.0002, *I*^2^ = 74%	Random-effects model	−1.11 [−1.42, −0.80]	7.10	0.00001
CRP	4	*P* = 0.04, *I*^2^ = 64%	Random-effects model	−1.50 [−2.00, −1.00]	5.91	0.00001
hs-CRP	2	*P* = 0.53, *I*^2^ = 0%	Fixed-effects model	−1.31 [−1.70, −0.93]	6.75	0.00001

*IL-6, interleukin-6; TNF-α, tumor necrosis factor-α; CRP, C-reactive protein; hs-CRP, high-sensitivity C-reactive protein*.

### Serum levels of CRP

Serum levels of CRP and hs-CRP were respectively mentioned in 4 studies (Wang, 2013; Ji et al., 2016; Li, 2016; Bian et al., 2017) and 2 studies (Shan, 2016; Chen et al., 2017). There were high heterogeneity among studies concerning CRP (*P* = 0.04, *I*^2^ = 64%) and no heterogeneity between studies concerning hs-CRP (*P* = 0.53, *I*^2^ = 0%), thus the random-effects model and fixed-effects model were adopted respectively. Statistically significant differences were observed in both CRP and hs-CRP levels between XBJ plus UTI group and single UTI group (SMD = −1.50, 95% CI [−2.00, −1.00], *P* < 0.00001; SMD = −1.31, 95% CI [−1.70, −0.93], *P* < 0.00001), which signified that XBJ combined with UTI decreased CRP levels in a greater degree than UTI alone (Table 2).

### LPS improvement

**Figure 8:**
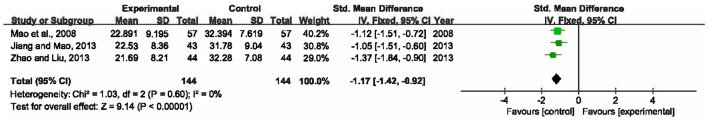
Forest plot of LPS improvement in sepsis patients treated with XBJ+UTI therapy and UTI alone.

The improvement of LPS was reported in 3 RCTs with 288 participants involved (Mao et al., 2008; Jiang and Mao, 2013; Zhao and Liu, 2013). No significant heterogeneity was detected among the trials (*P* = 0.60, *I*^2^ = 0%), and a fixed-effects model was utilized. The pooled analysis manifested that compared to single UTI, a combination of XBJ and UTI was more effective in lowering LPS levels (SMD = −1.17, 95% CI [−1.42, −0.92], *P* < 0.00001) (Figure 8).

### Publication bias

Limited by the small number of studies included in each outcome indicator (<10), we failed to assess publication bias by the means of carrying out a funnel plot. All included trials were published in Chinese academic journals. Since trials with negative or neutral results are less likely to be published, the efficacy of published studies might be overestimated. Consequently, the possibility of publication bias could not be ruled out.

### Sensitivity analysis

**Figure 9:**
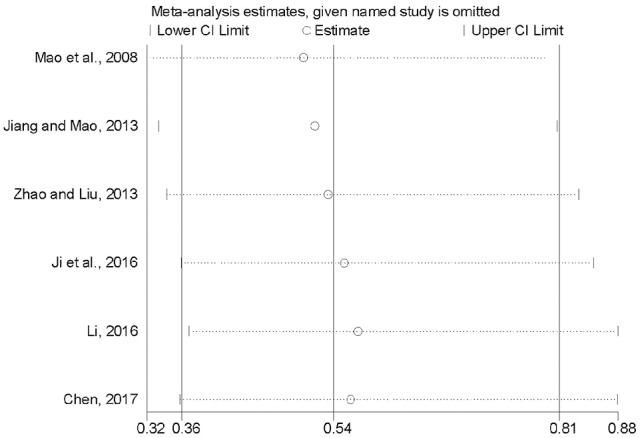
Sensitivity analysis of 28-days mortality.

To inspect the stability of the outcome, we implemented a sensitivity analysis of 28-day mortality (Mao et al., 2008; Jiang and Mao, 2013; Zhao and Liu, 2013; Ji et al., 2016; Li, 2016; Chen, 2017). By seriatim excluding one trial each time and re-performing meta-analysis of the remaining trials, we could observe whether the outcomes have dramatically changed. Figure 9 indicated that the outcomes of 28-day mortality were very similar, which had relatively good stability.

### Safety

Among all included studies, 7 RCTs definitely elucidated that no AE occurred in their treatment (Mao et al., 2008; Sun et al., 2010; Abuli et al., 2013; Wang, 2013; Li, 2015; Shan, 2016; Lu et al., 2017), 3 RCTs described 14 cases of AEs (Jiang and Mao, 2013; Zhao and Liu, 2013; Chen et al., 2017). Seven cases occurred in XBJ plus UTI group were manifested as 1 case of phlebitis, 4 cases of cutaneous pruritus, 1 case of mouth thirst, and 1 case of mild elevation of aminotransferase; while the other 7 cases associated with the administration of UTI were manifested as 2 cases of phlebitis, 2 cases of rash, 1 case of cutaneous pruritus, and 2 cases of nausea. All symptoms of AEs were slight, which could disappear after drug withdrawal (Jiang and Mao, 2013) or relieve after symptomatic treatment (Zhao and Liu, 2013; Chen et al., 2017). The rest 7 RCTs made no mention of AEs (Ye and Wu, 2010; Zhou and Fang, 2013; Ji et al., 2016; Li, 2016; Bian et al., 2017; Chen, 2017; Li and Jia, 2017).

## Discussion

Sepsis has been called a hidden public health disaster that tremendously threatens people's health and living quality. Patients who survive sepsis not only have to endure long-term cognitive impairment and physical disability, but also suffer a more-than-doubled risk of death in the following 5 years (Quartin et al., 1997; Angus, 2010; Iwashyna et al., 2010). International guidelines for sepsis management recommend the prompt identification of sepsis and broad-spectrum antibiotics therapy (Rhodes et al., 2017; Seymour et al., 2017). Clinical practice validates early treatment with appropriate antibiotics is an effective means for sepsis, by which patients' prognoses can usually be improved (Feng et al., 2015). However, the mis- and overuse of broad-spectrum antibiotic agents result in the emergence of multiple drug-resistant bacillus, antibiotics available for clinicians to select is extremely limited. Given the high morbidity, high mortality and poor prognosis of sepsis, it is critical to identify more effective, innovative, adjunctive therapeutic strategies and drugs for clinical application.

UTI is a promising drug for regulating patients' immune function, whose immunomodulatory property has been widely investigated for the treatment of sepsis. An Indian multicenter RCT indicated that severe sepsis treated with UTI was associated with a reduction in 28-day all-cause mortality (Karnad et al., 2014). Additionally, numerous Chinese RCTs also manifested UTI can lower 28-day mortality, improve inflammatory response, mitigate damages to vital organs, shorten duration of mechanical ventilation and length of hospital stay in patients with sepsis (Xiao et al., 2013; Zhou, 2017).

XBJ is the only kind of traditional Chinese medicine injection approved for the treatment of sepsis in China (Ma et al., 2009). As an injection prepared from five Chinese herbs, approximately 30 bioactive compounds of it were identified or tentatively characterized, which include tanshinol, hydroxysafflor yellow A, paeoniflorin, ferulic acid, senkyunolide I and so forth (Ma et al., 2013; Zuo et al., 2017). Pharmacological researches confirmed that XBJ has capacities of regulating inflammatory response, alleviating microcirculation, protecting endothelial cells, improving immune function, and fighting oxidative stress of sepsis patients (Zheng, 2015; Li et al., 2016). A recent published meta-analysis indicated that compared with conventional therapies, XBJ as adjunctive therapy for sepsis could significantly decrease APACHE II score, 28-day mortality, temperature, and serum levels of PCT, WBC, CRP, and NEU (Shi et al., 2017).

Despite both XBJ and UTI achieving satisfactory efficacy in treating sepsis, most systematic reviews reported either XBJ or UTI, rather than the combination of them. With respect to XBJ and UTI combination therapy, only an English abstract was searchable in English database, and it's excerpted from an article published in Chinese academic journal (Liu and Li, 2014). The objective of current study is to provide an English full-text to evaluate the efficacy and safety of XBJ and UTI combination therapy for sepsis.

According to the results of this meta-analysis, XBJ and UTI combination therapy had more notable influence than single UTI on sepsis patients, which was reflected in the following aspects: first of all, XBJ plus UTI had an advantage over UTI alone in lowering 28-day mortality, which is the crucial clinical and prognostic parameter for evaluating therapeutic effect of a treatment (Liu et al., 2017). Moreover, the combination therapy was highly superior to UTI alone in improving indexes that had direct correlations with sepsis patients' conditions, namely, shortening duration of mechanical ventilation and length of ICU stay, and ameliorating APACHE II score.

### Potential mechanisms

CRP is an acute phase reactive protein that increases rapidly when inflammation or tissue damage occurs, and is therefore frequently utilized as a biomarker to evaluate the severity of sepsis (Reinhart et al., 2012). PCT is produced ubiquitously in response to endotoxin or to mediators released in response to bacterial infections (Gogos et al., 2000). Second to CRP, PCT to date has become the most widely utilized biomarker in sepsis management worldwide. Both CRP and PCT can serve as significant indicators in evaluating therapeutic effects of sepsis. This meta-analysis exhibited that compared with single UTI, XBJ plus UTI was associated with dramatically lower PCT, CRP, and hs-CRP levels.

Inflammatory cytokines are immune-modulating products, whose secretion occurs from the very first moment of sepsis. Persistently high or increasing levels of inflammatory cytokines are mostly detected in non-survivors of sepsis, while the opposite are detected in survivors (Heper et al., 2006; Reinhart et al., 2012). In this systematic review, comparison with UTI revealed that XBJ combined with UTI could remarkably improve inflammatory cytokines by reducing IL-6 and TNF-α levels.

LPS released by invading bacteria is an early sign of infection. Minute amounts of it can result in fatal septic shock if inflammatory reaction is amplified and uncontrolled (Park et al., 2009). LPS concentration is a potential indicator of sepsis severity and treatment effect. The meta-analysis result suggested that XBJ combined with UTI had a more prominent performance on lowering LPS level than UTI.

### Safety

With regard to AEs, all their symptoms reported were mild and transient. No difference was shown in quantity of AEs between the two groups, both of which were 7 cases. Since phlebitis and cutaneous pruritus were detected in both groups, we suspect they might be attributable to the administration of UTI. To decrease the incidence of AEs, drugs should be applied strictly in accordance with instructions. Before initiating treatment, clinicians are recommended to carefully enquire whether patients have allergic history of relevant drugs. In addition to studies claiming no occurrence of AEs, there remained 7 studies that did not refer to AEs, consequently, a definite conclusion on the safety of XBJ and UTI combination therapy for sepsis can not be drawn from the provided information. In our further research, clinical trials focused specifically on safety should be synthesized to fully elucidate the safety of the combination therapy.

### Limitations

There were, of course, still some potential limitations that might downgrade the certainty of this paper. (1) Our research only retrieved studies published in English and Chinese, which might result in a certain degree of selective bias because no reference was made to studies published in other languages. (2) All included studies were conducted in China, therefore, whether the findings of our paper could be generalized to broad ranges of geography and ethnic origin was slightly in doubt. (3) All included trials stated random allocation was adopted, nevertheless, some of them did not elaborate on the means by which randomization were implemented. (4) Although blinding and allocation concealment are vitally important elements to ensure methodological quality of clinical trials, none of the original studies made adequate descriptions of these. The investigators and participants might have been aware of the therapeutic interventions implemented, which could lead to the emergence of false-positive conclusions. (5) Considerable heterogeneity among trials was detected in some outcome indicators. That measurement methods of the same outcome differed across the studies may be the source of heterogeneity. (6) The trials included primarily compared short-term 28-day mortality, while 2002 Brussels Roundtable, “Surviving Intensive Care”, highlighted that clinical trials should include long-term follow-up of survival rate and quality of life, and follow-up ought to be for at least 6 months (Angus and Carlet, 2003). Consequently, researches to gather further data of long-term prognoses are clearly needed.

## Conclusions

In summary, our study made a comprehensive comparison on efficacy and safety between XBJ plus UTI and UTI alone for sepsis. The findings provided evidence that the combination therapy had superiority over single UTI in improving short-term survival rate, alleviating illness severity, shortening ICU stay and mechanical ventilation duration, and decreasing PCT, inflammatory cytokines, CRP, and LPS levels. However, drawbacks of the included studies—small sample sizes and general methodological quality—may undermine the credibility of these findings, thus, we cautiously come to a conclusion that XBJ and UTI combination therapy was beneficial for sepsis. Our study here provides not only an evidence-based approach to novel therapy for sepsis, but also a framework for designing future preclinical and clinical trials. It is essential to carry out more rigorously designed, larger-scale, multicenter, higher-quality RCTs to further confirm our findings.

## Author contributions

GC conceived the study, conducted the database search, assessed studies for inclusion, extracted the data, and prepared the manuscript. YG searched the databases, assessed studies for inclusion, extracted the data followed by cross checking with GC. YJ, FY, SL, and DT analyzed the data. QM revised the manuscript.

### Conflict of interest statement

The authors declare that the research was conducted in the absence of any commercial or financial relationships that could be construed as a potential conflict of interest.

## Abbreviations

ACCP/SCCM: American College of Chest Physicians/Society of Critical Care Medicine
AEs: adverse events
APACHE II score: acute physiology and chronic health evaluation II score
CBM: Chinese Biomedical Database
CI: confidence intervals
CNKI: China National Knowledge Infrastructure
CRP: C-reactive protein
FDA: Food and Drug Administration
hs-CRP: high-sensitivity C-reactive protein
ICU: intensive care units
IL-6: interleukin-6
LPS: lipopolysaccharide
MD: mean difference
MODS: multiple organ dysfunction syndyome
PCT: procalcitonin
RCTs: randomized controlled trials
RR: risk ratio
SMD: standard mean difference
TNF-α: tumor necrosis factor-α
UTI: ulinastatin
VIP: China Science and Technology Journal Database
XBJ: Xuebijing injection.
